# Antiapoptotic Effect of Simvastatin Ameliorates Myocardial Ischemia/Reperfusion Injury

**DOI:** 10.1155/2013/815094

**Published:** 2013-12-19

**Authors:** Najah R. Hadi, Fadhil Al-amran, Maitham Yousif, Suhaad T. Zamil

**Affiliations:** ^1^Pharmacological Department, Medical College, Kufa University, Iraq; ^2^Cardiothoracic Surgical Department, College of Medicine, Kufa University, Iraq; ^3^Biology Department, College of Science, Al-Qadisiyah University, Al-Qadisiyyah, Iraq

## Abstract

*Background*. Myocardial ischemial reperfusion represents a clinically relevant problem associated with thrombolysis, angioplasty, and coronary bypass surgery. Injury of myocardium due to ischemial reperfusion includes cardiac contractile dysfunction, arrhythmias, and irreversible myocytes damage. These changes are considered to be the consequence of imbalance between the formation of oxidants and the availability of endogenous antioxidants in the heart. *Objective*. This study was undertaken to investigate the potential role of Simvastatin in the amelioration of myocardial I/R injury induced by ligation of coronary artery in a rat model. *Materials and Methods*. Adult male Swiss Albino rats were randomized into 4 equal groups. Group (1): sham group: rats underwent the same anesthetic and surgical procedures as those in the control group except ligation of LAD coronary artery, group (2): control group: rats were subjected to regional ischemia for 25 min and reperfusion for 2 hours by ligation of LAD coronary artery, group (3): control vehicle group: rats received vehicle of Simvastatin (normal saline) via IP injection and were subjected to regional ischemia for 25 min and reperfusion for 2 hours by ligation of LAD coronary artery, group (4): Simvastatin treated group: rats were pretreated with Simvastatin 1 mg/kg i.p. 1 hr before ligation of LAD coronary artery. At the end of experiment (2 hr of reperfusion), blood samples were collected from the heart for the measurement of plasma level of cardiac troponin I (cTnI). After that the heart was harvested and divided into 3 parts; one part was used for measurement of apoptosis, another part was homogenized for the measurement of tissue tumor necrosis factor-**α** (TNF-**α**), interleukin-1**β** (IL-1**β**), interleukin-6, monocyte chemoattractant protein-1, and macrophage inflammatory protein-1**α**, and the last part for histopathology study. *Results*. Compared with the sham group, levels of myocardial TNF-**α** and IL-1**β**, IL-6, MCP-1, and MIP-1**α** and plasma cTnI were increased (*P* < 0.05). Histologically, all rats in control group showed significant (*P* < 0.05) cardiac injury. Furthermore, all rats in control group showed significant (*P* < 0.05) apoptosis. Simvastatin significantly counteracted the increase in myocardium level of TNF-**α**, IL-1B, IL-6, MCP-1 and MIP-1**α**, plasma cTnI, and apoptosis (*P* < 0.05). Histological analysis revealed that Simvastatin markedly reduced (*P* < 0.05) the severity of heart injury in the rats that underwent LAD ligation procedure. *Conclusions*. The results of the present study reveal that Simvastatin may ameliorate myocardial I/R injury in rats via interfering with inflammatory reactions and apoptosis which were induced by I/R injury.

## 1. Introduction

Ischemia/reperfusion injury describes the experimentally and clinically prevalent finding that tissue ischemia with inadequate oxygen followed by successful reperfusion initiates a wide and complex array of inflammatory responses that may aggravate local injury as well as induce impairment of remote organ function [1]. Ischemial reperfusion injury results from several interdependent mechanisms, namely, oxidative stress, intracellular calcium overload and hypercontracture, endothelial cell activation with microvascular dysfunction, and altered myocardial metabolism [2]. Ischemial reperfusion injury prompts a release of oxygen free radicals, cytokines, and other proinflammatory mediators that activate both the neutrophils and the coronary vascular endothelium. Activation of these cell types promotes the expression of adhesion molecules on both the neutrophils and endothelium, which recruits neutrophils to the surface of the endothelium and initiates a specific cascade of cell-cell interactions, leading first to adherence of neutrophils to the vascular endothelium, followed later by transendothelial migration and direct interaction with myocytes. This specific series of events is a prerequisite to the phenotypic expression of reperfusion injury, including endothelial dysfunction, microvascular collapse and blood flow defects, myocardial infarction, and apoptosis [3]. I-R injury may occur in a variety of clinical settings, including reperfusion after thrombolytic therapy, coronary angioplasty, organ transplantation, aortic cross-clamping, or cardiopulmonary bypass [4]. Adaptive cellular responses activate the innate immune system with its Tall-like receptors and the complement system as well as the adaptive immune system. This results in a profound inflammatory tissue reaction with immune cells infiltrating the tissue. The damage is mediated by various cytokines, chemokines, adhesion molecules, and compounds of the extracellular matrix. The expression of these factors is regulated by specific transcription factor with NF-kB being one of the key modulators of inflammation [5]. Apoptosis is another mechanism of myocardial injury associated with ischemial reperfusion injury; it seems that apoptotic cell death in the myocardium is initiated during ischemia, but the energy needed for the execution is provided during reperfusion [6]. Cells undergoing apoptosis exhibit several typical features including shrinkage, cell membrane disruption, cytoskeleton rearrangement, nuclear condensation, and internucleosomal DNA fragmentation [7]. The degradation of DNA into fragments of approximately 185 bp in size with its multiples is one of the best characterized biochemical features of apoptotic cell death which is used as the basis for the commonly used labeling techniques for detecting apoptotic cells [8]. Simvastatin is member of statins that competitively inhibit the enzyme 3-hydroxy-3-methylglutaryl coenzyme A (HMG-CoA) reductase; the first committed step in cholesterol biosynthesis Simvastatin have pleiotropic (lipid lowering independent) encompass anti-inflammation, correction of endothelial dysfunction, increase in nitric oxide bioavailability, antioxidation, and stabilization of atherosclerotic plaques [9].

Naidu et al. (2003) demonstrated that after treatment with Simvastatin, the expression of NADPH oxidase is inhibited, which would result in a decrease in mitochondrial the transcription factors nuclear factor NF-*κ*Band activator protein (AP)-1 [10]. In addition, there are several mechanisms that have been proposed for statin-elicited beneficial effects, including the prevention of mevalonate formation and subsequently the synthesis of isoprenoid farnesyl pyrophosphate and geranylgeranyl pyrophosphate (GGPP), which leads to inhibition of the isoprenylation of small guanosine triphosphate-binding proteins, such as Rho or Ras proteins involved in cell differentiation, apoptosis, and inflammatory response [11].

Rajtík et al. (2012) found that HMG-CoA reductase inhibition by Simvastatin given orally for 5 days before induction of IRI is associated with abolishment of apoptotic cell death [12].

## 2. Materials and Methods

### 2.1. Animals

Sixty-six adult male Swiss Albino rats weighing 180–220 g were purchased from Animal Resource Center, the National Center for Drug Control and Researches. The animals were apparently healthy and they were housed in the Animal House of the College of Medicine/University of Kufa in a temperature-controlled (24 ± 2°C) room with ambient humidity and alternating 12 h light/12 h dark cycles and were allowed free access to water and standard chow diet until the start of experiments. The rats were left for two weeks without interference for acclimatization. They had no manifestation of any illness upon examination.

### 2.2. *In Vivo* Myocardial I/R Model

The *in vivo* myocardial I/R model was modified from a previous study [13]. Briefly, rats were anesthetized with 100 mg/kg ketamine and 5 mg/kg xylazine [14]. The rats were intubated and mechanical ventilation is then achieved by connecting the endotracheal tube to scientific ventilator (Harvard Model) at a respiratory rate of 138 breath/minute with a tidal volume of 20 mL/kg body weight [15]. A left thoracotomy was carried out to expose the heart. The LAD is then transiently ligated (or can be tied with a slipknot) using a 6-0 polypropylene suture for a 25-minute ischemic period [16]. After a 25 min ischemia, by microsurgical scissors that are used to cut the knot in the ligature (or by releasing the slipknot) the heart was reperfused for 2 h. Immediately after finishing the reperfusion time the rat was sacrificed, starting by injection of high dose from ketamine and xylazine, and after giving good time for in size the animal to go into deep anesthesia, the rat is positioned and the chest is opened in flap like manner revealing the heart; then a needle of the syringe is introduced into right ventricle to aspirate around 2 mL of blood for later plasma analysis. After that hearts were rapidly removed for quantification of myocardial injury, apoptosis, and biochemical studies [17].

### 2.3. Experimental Groups and Protocols

After the two weeks of acclimatization the rats were randomized into 6 groups, 6 rates in each group as follows.

*Sham Group*. Rats underwent the same anesthetic and surgical procedures but without ligation of (LAD) coronary artery.

*Control Group*. Rats underwent myocardial ischemia for 25 minutes and reperfusion for 2 hr, by ligation of (LAD) coronary artery. 

*Control Vehicle Group*. Rats pretreated with normal saline (vehicle for Simvastatin) via IP rout and underwent myocardial ischemia for 25 minutes and reperfusion for 2 hr, by ligation of LAD coronary artery.

*Simvastatin Treated Group*. Rats pretreated with Simvastatin 1 mg/kg [18] at 1 hr before ligation of LAD coronary artery via intraperitoneal injection [19]. Simvastatin was dissolved in normal saline [19] and given in a dose (1 mg/kg) via IP route at 1 hr before occlusion of LAD; Simvastatin was prepared immediately before injection.

### 2.4. Blood Sampling for Measurement of Plasma cTnI

At the end of reperfusion the blood was collected from the apex of heart; about 2 mL of blood was collected from the heart. The blood sample was placed in a tube containing disodium EDTA (22 mg/mL) as anticoagulant and mixed thoroughly then centrifuged at 3000 rpm for 15 min. Then it is used for the determination of plasma cTnI.

### 2.5. Tissue Preparation for Measurement of (TNF-*α*, IL-1B, IL-6, MCP-1, and MIP-1*α*)

Cardiac tissues collected 120 minutes after reperfusion were homogenized in a solution containing 1 : 10 (w/v) phosphate buffered saline that contains 1% triton X-100 and protease inhibitor cocktail [20], by using high intensity liquid processor. After homogenization, samples were centrifuged at 14,000 rpm for 20 min at 4°C [21]; the supernatant was collected and used in TNF-*α*, IL-1B, IL-6, MCP-1, and MIP-1*α* measurements using commercially available ELISA kits (signosis) according to the manufacturer's instructions.

### 2.6. Histopathological Analysis and Damage Score

Tissues were fixed in 4% paraformaldehyde and embedded in paraffin. Sections were stained with Haematoxylin and Eosin for histological evaluation of tissue damage. In order to have a quantitative estimation of cardiac damage, sections (*n* = 6 for each animal) were scored by 2 independent observers blinded to the experimental protocol. The following morphological criteria were considered: score 0, no damage; score 1 (mild), interstitial edema and focal necrosis; score 2 (moderate), diffuse myocardial cell swelling and necrosis; score 3 (severe), necrosis with the presence of contraction bands and neutrophil infiltrate; and score 4 (highly severe), widespread of necrosis with the presence of contraction bands, neutrophil infiltrate, and hemorrhage.

### 2.7. Determination of Myocardial Apoptosis

Myocardial apoptosis was based on the staining of condensed chromatin in apoptotic nuclei. since chromatin condensation in compact masses is the most specific and definite hallmark of apoptosis. The procedure is based on the selective denaturation of DNA in apoptotic cells by formamide and detection of denatured DNA with monoclonal antibody to single-stranded DNA (ssDNA). Formamide is a gentle agent that denatures DNA in apoptotic cells but not in necrotic cells or in the cells with DNA breaks in the absence of apoptosis.

The sensitivity of DNA in apoptotic cells to formamide is not related to DNA breaks but rather reflects changes in chromatin associated with apoptosis, such as chromatin condensation and digestion of proteins stabilizing DNA. The assay includes attachment of cells to 96-well plates, treatment of attached cells with formamide, and staining of ssDNA in apoptotic cells with a mixture of primary antibody and peroxidase-labeled secondary antibody. The protocol based on the one-step detection of ssDNA with antibody mixture has higher sensitivity and lower number of steps than standard two-step immunostaining.

This mixture is included in the kit in a ready to use form. The detection of apoptotic cells with formamide-mAb procedure is based on the staining of condensed chromatin in apoptotic nuclei. Since chromatin condensation in compact masses is the most specific and definite hallmark of apoptosis, this method provides universal detection of apoptosis. The apoptotic events that occur without DNA breaks or without activation of specific caspases will be detected with our mAb to ssDNA.

### 2.8. Statistical Analysis

Statistical analyses were performed using SPSS 20.0 for windows.lnc. Data were expressed as mean ± SEM. Analysis of variance (ANOVA) was used for the multiple comparisons among all groups followed by post hoc tests using LSD method. The histopathological grading of heart changes is a nonnormally distributed variable measured on an ordinal level of measurement; therefore, nonparametric tests were used to assess the statistical significance involving this variable. The statistical significance of difference in total score between more than 2 groups was assessed by Kruskal-Wallis test, while Mann-Whitney *U* test was used for the difference between 2 groups. In all tests, *P* < 0.05 was considered to be statistically significant.

## 3. Results

### 3.1. Simvastatin Reduced Myocardial TNF-*α*, IL-1B, and IL-6 following MI/R Injury

**Figure 1:**
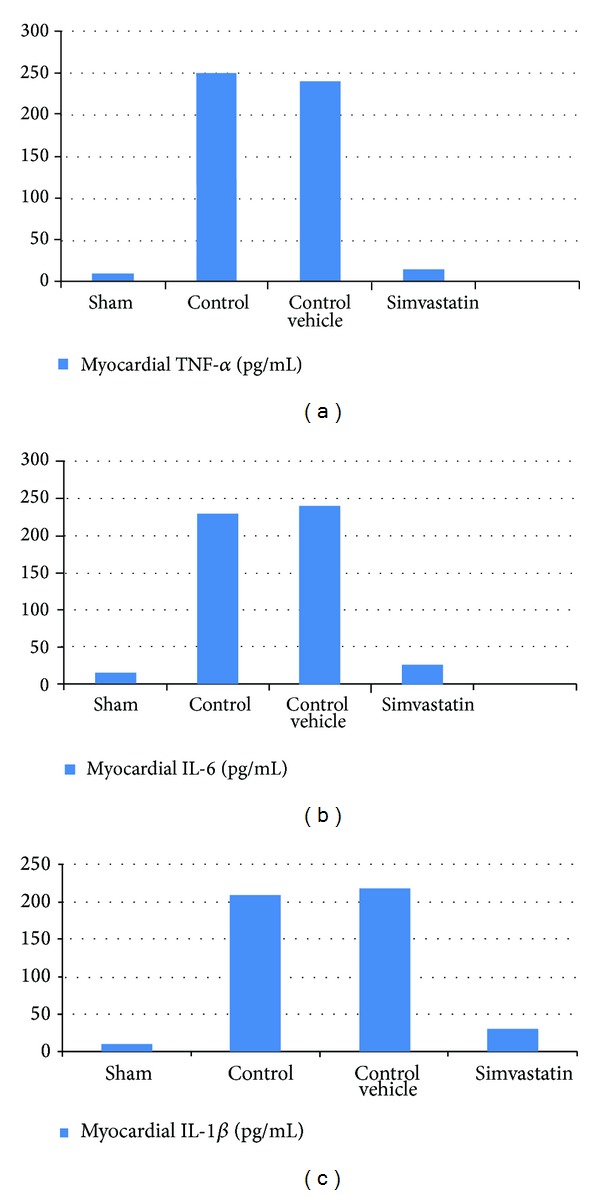
The mean of (a) myocardial TNF-*α* level (pg/mL), (b) myocardial IL-6 level (pg/mL), and (c) Myocardial IL-1*β* (pg/mL) in the six experimental groups at the end of the experiment.

Myocardium levels of inflammatory cytokines following MI/R were analyzed by ELISA. Figures 1(a), 1(b), and 1(c) show that MI/R injury increased significantly (*P* < 0.05) the levels of myocardium of TNF-*α*, IL-1B, and IL-6 compared with the sham group (*P* < 0.05). In the Simvastatin treatment group, myocardium levels of TNF-*α*, IL-1B, and IL-6 were reduced significantly compared with the control group (*P* < 0.05).

### 3.2. Simvastatin Reduced the Myocardial MCP-1 and MIP-1*α* following MI/R Injury

**Figure 2:**
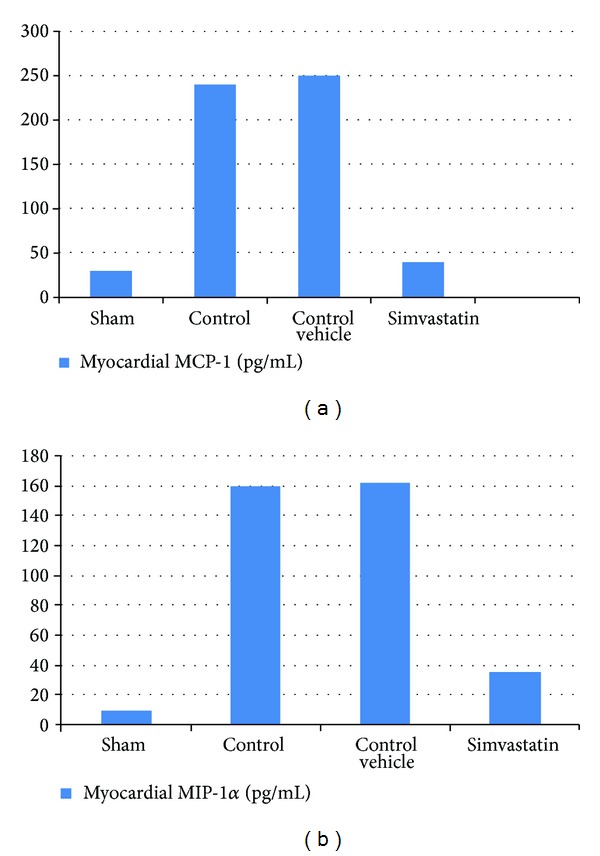
The mean of (a) myocardial MCP-1 level (pg/mL) and (b) myocardial MIP-1*α* level (pg/mL) in the six experimental groups at the end of the experiment.

Myocardium levels of inflammatory chemokines following MI/R were analyzed by ELISA. Figures 2(a) and 2(b) show that MI/R injury increased significantly (*P* < 0.05) the levels of myocardial MCP-1 and MIP-1*α* compared with the sham group (*P* < 0.05). In the simvastatin treatment group, myocardium levels of MCP-1 and MIP-1*α* were reduced significantly compared with the control group (*P* < 0.05).

### 3.3. Histopathological Findings

**Figure 3:**
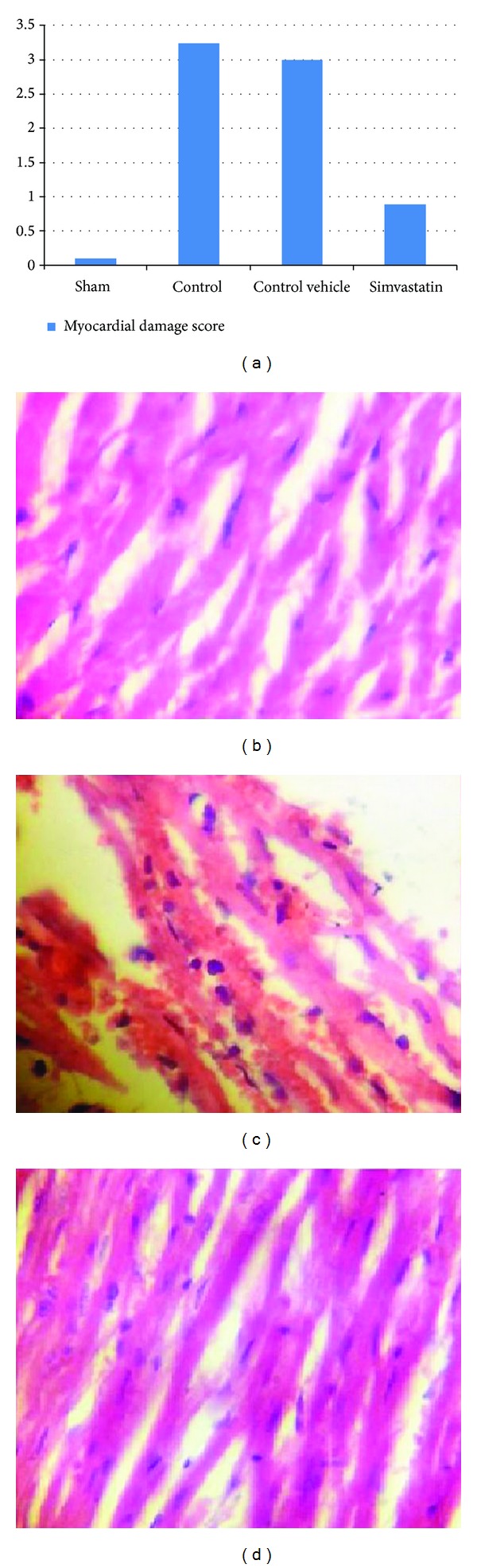
(a) shows the difference in mean ± SEM values of total severity scores in the six experimental groups. (b) Photomicrograph of heart section of normal rats shows the normal architecture. The section is stained with Haematoxylin and Eosin (×10). (c) Photomicrograph of cardiac section for the control vehicle group showed interstitial edema, hemorrhage, and PMN infiltration. The section is stained with Haematoxylin and Eosin (×40). (d) Photomicrograph of cardiac section in Simvastatin treated group. The section showed almost normal cardiac tissue; the section is stained with Haematoxylin and Eosin (×40).

Treatment of rats with Simvastatin improved cardiac injury significantly (*P* < 0.05) as compared with control vehicle group and the total severity scores mean of this group showed that 16.7% of the group had no damage, 66.7% had mild cardiac injury, and 16.7% had moderate cardiac injury. A cross-section of sham rat's heart showed a normal cardiac structure. All rats in this group showed 100% normal hearts as shown in Table 1. There was statistically insignificant difference between control vehicle group (III) and control group (II) (*P* > 0.05) and the total severity scores of the control group showed that 16.7% of the group had moderate cardiac injury, 66.7% had severe cardiac injury, and 16.7% had highly severe cardiac injury (Figures 3(a), 3(b), 3(c), and 3(d)).

### 3.4. Simvastatin Alleviated Myocardial Apoptosis following MI/R Injury

**Figure 4:**
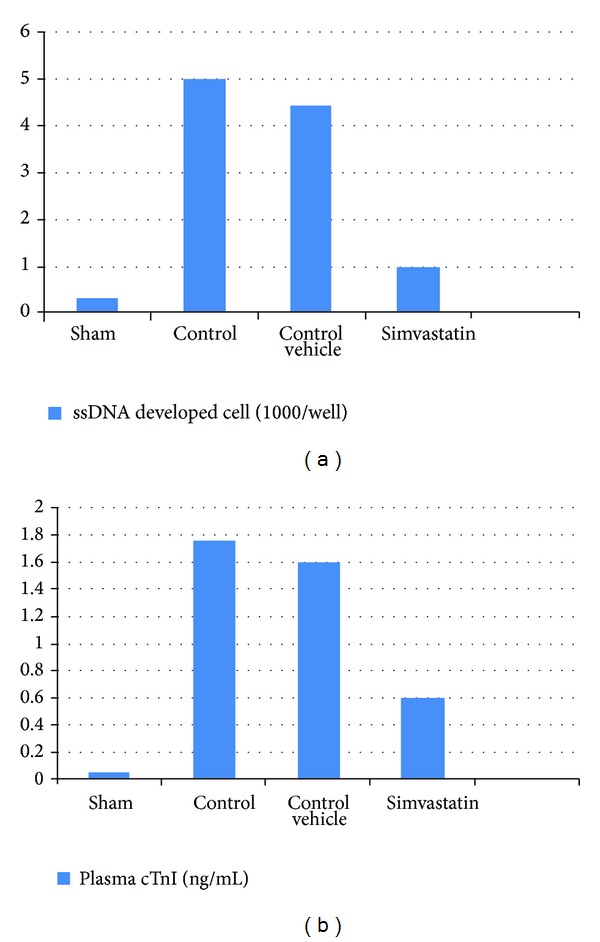
The mean of (a) ssDNA fragmentation (ssDNA developed cell ∗ 1000/well) and (b) plasma cTnI level (ng/mL) in the six experimental groups at the end of the experiment.

Substantial evidence suggests that apoptosis plays a critical role in cardiomyocyte loss and subsequent development of cardiac dysfunction after MI/R injury; the level of myocardial ssDNA fragmentation significantly (*P* < 0.05) increased in induced untreated (control) group as compared with sham group. The level of myocardial ssDNA fragmentation significantly (*P* < 0.05) decreased in the Irbesartan treated group as compared with control group (Figures 4(a) and 4(b)).

### 3.5. Simvastatin Reduced Cardiac Troponin I following MI/R Injury

See Figure 4.

## 4. Discussion

The major findings of the present study are as follows. Firstly, the inflammatory cytokine (TNF-*α*, IL-1B, and IL-6), cc chemokines (MCP-1, MIP-1*α*), and apoptosis play important role in the pathology of myocardial I/R. Secondly, Simvastatin pretreatment played a protective role against myocardial I/R injury; the protective effects of Simvastatin during myocardial I/R injury were correlated with the attenuation of inflammation and apoptosis. Thirdly, Simvastatin ameliorated myocardial I/R injury as evidenced by reducing the release of cardiac specific enzyme troponin I and myocardial damage.

Coronary arterial occlusion due to thrombosis after atherosclerotic plaque rupture is the major cause of myocardial infarction. These acute events represent the leading cause of death worldwide. Early reperfusion is the best method to salvage the ischemic organ; however, it leads to additional damage known as reperfusion injury [22]. The early reperfusion phase is characterized by enhanced release of ROS from endothelial cells and cardiomyocytes, as well as enhanced expression of cytokines and adhesion molecules. The enhanced expression of chemokines during the first hours of reperfusion triggers further recruitment of neutrophils and monocytes into the infracted myocardium which lead to increasing the cardiac damage by further releasing ROS, inflammatory mediators, and proteases [23].

Zhang et al. (2005a) showed that Simvastatin markedly attenuated the production of TNF-*α*, IL-1*β*, IL-6, and increased IL-10 levels in the noninfarcted and infarcted regions, reduced collagen deposition in the noninfarcted myocardium, and improved left ventricular function [24]. Furthermore, Sheng et al. (2009) found that Simvastatin markedly inhibits the expression of TLR4, TNF-*α*, and IL-6 in the myocardium after MI [25]. Hajipour et al. (2009) showed that Simvastatin pretreatment reduced intestinal I/R injury and was associated with downregulation of serum TNF-*α* and tissue malondialdehyde level and Simvastatin administration maintained cellular antioxidant enzyme contents compared to the I/R group after 3-hour reperfusion time [26]. Dantas et al. (2010) found that Simvastatin pretreatment attenuated cyclophosphamide-induced urothelium inflammation in an experimental rat model, through significantly reducing plasma level of proinflammatory cytokines (TNF-*α*, IL-6, and IL-1*β*) [27].

Veillard et al. (2006) showed that Simvastatin inhibits the expression of the chemokines MCP-1, MIP-1*α*, and MIP-1*β* and the chemokines receptors CCR1, CCR2, CCR4, and CCR5 at the mRNA level in human ECs and macrophages via inhibition of the geranylgeranyl pyrophosphate pathway [28]. Bruegel et al. (2006) suggest that statin-mediated immunomodulation by inhibiting MIP-1*α* could contribute to the beneficial effects of statin therapy independent of lowering plasma cholesterol [29]. Moreover, Cakmak et al. (2012) showed that statin decreased MCP-1 expression in an in-cultured endometriotic cells [30].

Yang et al. (2009) demonstrated that Simvastatin improved cardiac function and suppressed serum cTnI in the patients with CHF [31]. Almansob et al. (2012) showed that Simvastatin was administrated for patient undergoing noncoronary artery cardiac surgery; Simvastatin significantly reduced plasma troponin T, isoenzyme of creatine kinase, C-reaction protein, blood urea nitrogen, creatinine, interleukin-6, and interleukin-8 [32]. Veselka et al. (2006) found that pretreatment with statins in patients undergoing PCI for stable angina pectoris reduces the risk and extent of procedure-related myocardial injury measured by troponin release [33]. Abd Elbaky et al. (2010) studied the possible protective effect of Simvastatin (SIM), against doxorubicin-induced cardiotoxicity. They found that rats that received simvastatin alone showed apparently normal myocardial features similar to those of normal control, while rats administered doxorubicin showed typical myocardial toxicity in a form of myocardial muscle coagulative necrosis with focal areas of fibrosis, vascular dilatation and congestion, valves edema, and massive mononuclear cellular infiltration. Meanwhile, rats that received doxorubicin and were pretreated with simvastatin showed few inflammatory cells infiltration, little edema, and improvement of myocardial cell necrosis [34]. Statin mediated the inhibition of Rho kinase leading to activation of phosphatidylinositol-3 kinase (PI3 K)/protein kinase Akt pathway that promotes the survival of the myocardial tissue [35].

Slijper et al. (2010) found that treatment with simvastatin resulted in a significant decrease in cell apoptosis rate and prevented gut mucosal damage following intestinal IR in a rat [36]. Dibazar et al. (2008) published similar conclusions regarding lung and liver injuries after ischemial reperfusion injury, attributing this positive effect of simvastatin to inhibition of inflammation and apoptotic pathway [37]. Ko et al. (2011) found that intravitreal injection of simvastatin immediately following reperfusion in retinal I/R injury led to an increase in the level of antiapoptotic protein Bcl-2 ischemic retinas. In contrast, the level of the proapoptotic protein Bax was not affected by simvastatin treatment. Therefore, this increased Bcl-2/Bax ratio might have contributed to decreased retinal neuron degeneration following ischemia [38].

## Figures and Tables

**Table 1:** The differences in histopathological scoring of abnormal heart changes among the four experimental groups.

	Sham		Control		Control vehicle		Simvastatin	
Normal	6	100%	0	0	0	0	1	16.7%
Mild	0	0	0	0	0	0	4	66.7%
Moderate	0	0	0	0	1	16.7%	1	16.7%
Severe	0	0	4	66.7%	4	66.7%	0	0
Very severe	0	0	2	33.3%	1	16.7%	0	0

Total	6	100%	6	100%	6	100%	6	100%
